# Physical Exercise and Quality of Life among Healthcare Professionals

**DOI:** 10.1192/j.eurpsy.2026.10760

**Published:** 2026-07-31

**Authors:** M. Theodoratou, C. Mega, M. Argyrides, C. Koundourou, G. A. Kougioumtzis

**Affiliations:** 1 Social Sciences, Hellenic Open University, Patras, Greece; 2 Health Sciences, Neapolis University Pafos, Pafos; 3 Kapodistrian and National University of Athens, Athens, Cyprus

## Abstract

**Introduction:**

Healthcare professionals experience high levels of occupational stress, which can adversely impact their physical and psychological well-being. Physical activity is known to contribute positively to health, stress reduction, and quality of life, yet limited research has examined these associations within the Greek healthcare workforce. Despite this evidence, the degree to which physical activity is practiced by healthcare professionals themselves and its potential association with their quality of life has not been sufficiently investigated in Greece. This issue is particularly relevant, as healthcare workers are simultaneously caregivers and individuals whose well-being is central to the effectiveness of healthcare systems.

**Objectives:**

This study aimed to assess the level of physical activity among healthcare professionals in the 6th Regional Health Authority of Greece and to explore its relationship with perceived quality of life.

**Methods:**

A cross-sectional study was conducted with 77 healthcare professionals using an online questionnaire. Data collection instruments included a demographic survey, the International Physical Activity Questionnaire (IPAQ), and the World Health Organization Quality of Life Questionnaire (WHOQOL-BREF). Statistical analysis was performed with IBM SPSS 27, employing descriptive statistics, correlation analyses (Pearson and Spearman), and non-parametric tests (Kruskal–Wallis), with significance set at p < 0.05.

**Results:**

Participants were predominantly women (90.9%) with a mean age of 39.6 years, and nurses constituted the majority (63.6%). IPAQ findings indicated low engagement in vigorous activity (mean 1.6 days/week, ~45 minutes), while moderate activity (2 days/week, ~82 minutes) and walking (4.6 days/week, ~55 minutes) were more common. Sedentary behavior averaged 338 minutes daily. WHOQOL-BREF scores revealed generally satisfactory physical, psychological, and environmental quality of life. No significant correlations emerged between physical activity and quality of life dimensions. However, education level significantly influenced quality of life, with higher education linked to better quality of life outcomes.

**Conclusions:**

This study provides insight into the physical activity behaviors and quality of life perceptions of healthcare professionals in the 6th Regional Health Authority of Greece. Although physical activity is recognized as beneficial for both physical and mental health, the present findings did not identify a direct statistical relationship between activity levels and quality of life within this sample. Instead, education appeared to play a more decisive role, suggesting that higher levels of academic training and ongoing professional development may enhance overall well-being. These results highlight the importance of supporting healthcare staff through continuing education programs and workplace interventions that foster both professional growth and personal health.

**Disclosure of Interest:**

None Declared

